# Infant and Pre-birth Involvement With Child Protection Across Australia

**DOI:** 10.1177/10775595231186647

**Published:** 2023-06-29

**Authors:** Melissa O’Donnell, Fernando Lima, Miriam Maclean, Rhonda Marriott, Stephanie Taplin

**Affiliations:** 1 Australian Centre for Child Protection, 613331 University of South Australia, Perth, WA, Australia; 2 Telethon Kids Institute, Nedlands, WA, Australia; 3 Ngangk Yira Institute for Change, 5673 Murdoch University, Perth, WA, Australia; 4 School of Public Health, Faculty of Health, 110561 University of Technology Sydney, Sydney, NSW, Australia

**Keywords:** infants, child maltreatment, child protective services, longitudinal research, path analysis, quantitative research

## Abstract

Infants (<1 year old) are the age group in Australia with the highest rate of involvement with child protection. Many jurisdictions across Australia and internationally are implementing policies focused on prenatal planning and targeted support. This study investigates Australian trends in prenatal and infant child protection notifications, substantiations and out-of-home care; and the extent of over-representation of Aboriginal and Torres Strait Islander infants. Data was provided by the Australian Institute of Health and Welfare for the period 1 July 2012-30 June 2019. Univariate Poisson regression analysis was conducted, reporting the percentage change in the incidence rate ratios. All Australian jurisdictions who collect and approved release of prenatal notification data experienced increases in the rates of children with prenatal notifications, with a 4% (IRR: 1.04(1.04–1.05)) overall increase per year across Australia. Approximately 33% of children had substantiated prenatal notifications. Rates of infant notifications and entry to care in Australia increased overall by 3% (IRR:1.03(1.03–1.04)) and 2% per year (IRR:1.02(1.01–1.03)), respectively. With rising numbers of families reported prenatally and during infancy, greater evidence of the effectiveness of policies, interventions and outcomes for children and families is required.

## Introduction

In Australia and internationally we are seeing an increase in families being reported to statutory child protection services during pregnancy and following birth (Australian Institute of Health and Welfare, 2021; Pearson et al., 2020). Several studies in the UK have highlighted the increase in the number of children in contact with the child protection system at a younger age, with a large proportion involved with the system at or prior to birth (Broadhurst et al., 2015; McGhee et al., 2017). In the United States there has also been federal statute changes to the Child Abuse Prevention and Treatment Act which specifies requirements for child welfare agencies to receive notifications of infants prenatally substance exposed and to provide support through Plans of Safe Care programs (Deutsch et al., 2022).In Australia, infants (children aged <1 year) are now the age group with the highest rate of notification to child protection services, substantiated maltreatment and entry into out-of-home care (OOHC) (Australian Institute of Health and Welfare, 2021). This increased child protection involvement with the youngest subset of children has been attributed to increased awareness of several issues: 1) a general recognition of the importance of early intervention across many areas of social and health policy; 2) the extreme physical and developmental vulnerability of infants and; 3) the potential for harm during pregnancy resulting in infants born with neonatal withdrawal syndrome or foetal alcohol spectrum disorder (Juhasz, 2020). Many jurisdictions across Australia and internationally have implemented policies to facilitate reporting and responding to child protection concerns raised prenatally (Critchley, 2018; McGhee et al., 2017). The aim of prenatal reporting is to allow early support and intervention that may prevent infant entry into out-of-home care and identify cases where infant removals are considered necessary (Taplin, 2017).

Prenatal notifications may occur when there are concerns held for an unborn baby due to family violence, mental health issues or drug and alcohol use. Child protection concerns in a family where other children have been removed due to risk of significant harm or abuse and neglect (the negligent treatment of a child to an extent that can affect their development or cause an injury) may also result in prenatal notifications (Department of Communities, 2021). There is evidence that an increasing rate of substance use and domestic violence during pregnancy is one of a number of factors contributing to the rise in pre-birth and infant involvement in child protection (Davies et al., 2016). Some studies have identified a number parental or family factors (e.g.: maternal age, maternal education, marital status), as well as socio-economic factors (e.g: housing, parental employment) to be associated child protection involvement, both pre and postnatally (Baldwin et al., 2020). However it is often a combination of these issues, which contributes to a risk of maltreatment and entry into out-of-home care (Baldwin et al., 2020; Fuller-Thomson et al., 2019). Families with an infant removed and placed into out-of-home care often have three or more risk factors, and commonly a child protection history for parents and/or older siblings (Meiksans et al., 2021)

Aboriginal and Torres Strait Islander families are over-represented in child protection systems, with previous research reporting on the community concerns arising from the increase in Aboriginal and Torres Strait Islander infants removed from their families (O’Donnell et al., 2019). Aboriginal and Torres Strait Islander people are Australia’s First People and represent approximately 4% of Australia’s population with one-third of the Aboriginal and Torres Strait Islander population under 15 years of age (ABS, 2021). There are a number of factors that contribute to the disproportionate representation of Aboriginal and Torres Strait Islander families who have contact with statutory child protection system including poverty, colonisation and intergenerational trauma from forced removals (AIFS, 2020). Given that Aboriginal and Torres Strait Islander children are receiving child protection services at a national rate eight times that of non-Aboriginal and Torres Strait Islander children, it is important to determine whether this is a uniform pattern across Australia (AIHW, 2021). Reducing the over-representation of Aboriginal and Torres Strait Islander children in the child protection system is now a target of the Australian Government’s *Closing the Gap* strategy, with the aim to reduce the national over-representation of Aboriginal and Torres Strait Islander children in out-of-home care by 45% by 2031 (Commonwealth of Australia, 2020).

Similar to the UK there is variation across Australian jurisdictions, with each state having their own child protection jurisdiction and therefore different policies and practices in regard to the prenatal reporting of families and child protection processes which occur pre- and post-birth (Bunting et al., 2017). In most jurisdictions parents can be reported to child protection prenatally. The AIHW refer to these reports as “unborn child” reports, however, in states such as New South Wales (NSW) and the Australian Capital Territory (ACT) they are referred to as prenatal reports, the term which will be used in this study. There is variability in state responses to these reports. In Victoria, for example, the notification is not classified as a protective intervention report so there can be no investigation, substantiation of the report or protective intervention prior to birth (Victorian Health and Human Services, 2021a). Victoria therefore do not collect prenatal report data which can be compared to the other jurisdictions. However, all Australian state and territory child protection jurisdictions have data for infants with reports, investigations, substantiations, and entry into out-of-home care.

This study is part of a larger research program examining the nature, extent and impacts of infant removals (*State Interventions with Babies*) in Australia. This specific study aims to:

(i) determine whether a uniform increase or decrease over time in children with child maltreatment reports and substantiations, both prenatally and for infants, across Australia is evident.
(ii) determine whether there are changes across jurisdictions in the timing of infant removals, whether they are being removed earlier now than in the recent past.
(iii) quantify the extent to which there is an over-representation of Aboriginal and Torres Strait Islander infants entering care across the jurisdictions.
(iv) examine whether there is an association between prenatal reporting and infant removals across jurisdictions.
(v) extend the knowledge about similarities and differences across jurisdictions in child protection involvement prenatally and in the first year.

A greater insight into the patterns of involvement by each jurisdiction’s child protection system in the lives of pregnant women^1^ and their infants will allow for improved policy responses and more targeted preventions strategies within and beyond Australia.

## Methods

### Data Source

This study uses data provided from the Australian Institute of Health and Welfare’s (AIHW) National Child Protection Minimum Dataset for the period 1 July 2012 to 30 June 2019. Across Australia, the eight state and territory child protection departments collect data at a unit record level (child level) with all jurisdictions, except New South Wales (NSW) who provides aggregated data, providing data to the AIHW according to national agreed definitions and technical specifications.

### Child Protection Data

The AIHW dataset counts each child’s involvement with child protection only once during each period of yearly reporting (e.g. if a child received more than one notification during 2017-18 then they are only counted once in the notifications during that period). The data provided for the study includes prenatal notifications (i.e. women^1^ reported to child protection during pregnancy) and notifications about infants. A notification within Australia is a report made to a child protection department alleging child abuse/neglect, child maltreatment or harm to a child (Australian Institute of Health and Welfare, 2020). A substantiation is the result of a finalised investigation which concludes that there is reasonable cause to believe that a child has been, is being or is likely to be, abused, neglected or otherwise harmed (AIHW, 2020). Notifications and substantiations can occur for infants in all jurisdictions, but not all jurisdictions report notifications and substantiations prenatally before birth (Northern Territory -NT- reported nil and Victoria -Vic- does not report prenatal notifications and substantiations). In relation to the dataset used in this study, NSW, Western Australia (WA), and Queensland (Qld) have authorised the reporting of their individual state data, but South Australia (SA), Tasmania (Tas) and the Australian Capital Territory (ACT) have only authorised the use of their data in a combined total, not disaggregated by jurisdiction due to the small numbers and risk of disclosure.

Out-of-home care (OOHC) is used in Australia where a child is removed from their family and placed in an alternative living arrangement due to child safety concerns. Study data include the total number of children in OOHC as well as those who are admitted to and discharged from care during the reporting period. The primary type of maltreatment for prenatal and infant substantiations are reported including physical abuse, emotional abuse, sexual abuse or neglect.

Due to the disproportionate representation of Aboriginal and Torres Strait Islander children and families involved in the child protection system, this paper has reported specific Aboriginal and Torres Strait Islander rates across Australia and by states. Total child numbers and rates were included instead of non-Aboriginal and Torres Strait Islander as there were a large group of children with unknown Aboriginal and Torres Strait Islander status.

In Australia, each state and territory governments are responsible for statutory child protection. Different child protection policies and data collection processes in each jurisdiction may result in differences in the number of children involved with the child protection system and/or their level of involvement (notifications, substantiations and/or OOHC placements) reported by each state and territory. This study is focussed on the number of children reported by each jurisdiction as involved in their child protection system and this is used as a unit of measure to investigate trends over time for prenatal and infant’s child protection involvement for each state and territory, with an aggregated total for all states.

### Presentation of Numbers and Rates of Children Involved in Child Protection

Prenatal notifications and substantiations are reported for NSW, WA and Qld, and an aggregated count for SA, ACT and Tas (note that NT, Vic and SA do not have legislation for recording prenatal notifications and NT, Vic, ACT and SA don’t conduct investigations pre-birth). From 2014-15, Qld data are sourced from the Child Protection National Minimum Data Set according to nationally determined definitions and technical specifications, therefore may not be comparable to previous years or match figures published elsewhere. NSW has changed their client system from 2017-18, with missing records for substantiations for that period and available again for 2018-19 under the new client system. SA, Tas and ACT have only authorised the presentation of an aggregated count over the whole period under study to avoid identification risks. Notifications and OOHC placements for infants are reported for NSW, Vic, Qld, WA and an aggregated count for SA, ACT, Tas and NT to avoid identification risks.

Rates per 1000 children per year (report period) were calculated for each of the indicators under analysis. For prenatal notifications and substantiations, rates were calculated using children with prenatal notifications/substantiations as the numerator and the population of live births during the reporting period as the denominator (Australian Bureau of Statistics, 2019; 2020). Similarly, notifications and care placements of infant (aged <1 year) rates were calculated using children with notification/care placement as infants as the numerator and the population of infants aged <1 year during the reporting period as the denominator. All rates are presented per 1000 children to describe the number children with child protection contacts (notification, substantiations, or care placements) that occurred in a population per 1000 people (e.g. infants). NSW was the most populated jurisdiction in our study with 98,580 children under 1 year of age in 2018-19, followed by Vic with 78,132 infants, Qld with 59,881 infants, WA with 32,848 and the combined SA, ACT, Tas and NT with 33,181 infants (Australian Institute of Health and Welfare, 2020).

### Trend Analysis

To investigate change over time, a trend analysis was conducted using univariate Poisson regression models. A separate analysis was conducted for each jurisdiction and for the three different totals described before. The percentage change in the incidence rate ratios (IRRs) and 95% confidence intervals are presented as an indication of trends in prenatal notifications and substantiations, and in notifications and care placements for infants over the period 2012-13 to 2018-19. IRRs are described in the main body of this study and a table with all results are presented in the Appendix.

## Results

### Prenatal Notifications

**Figure 1.**
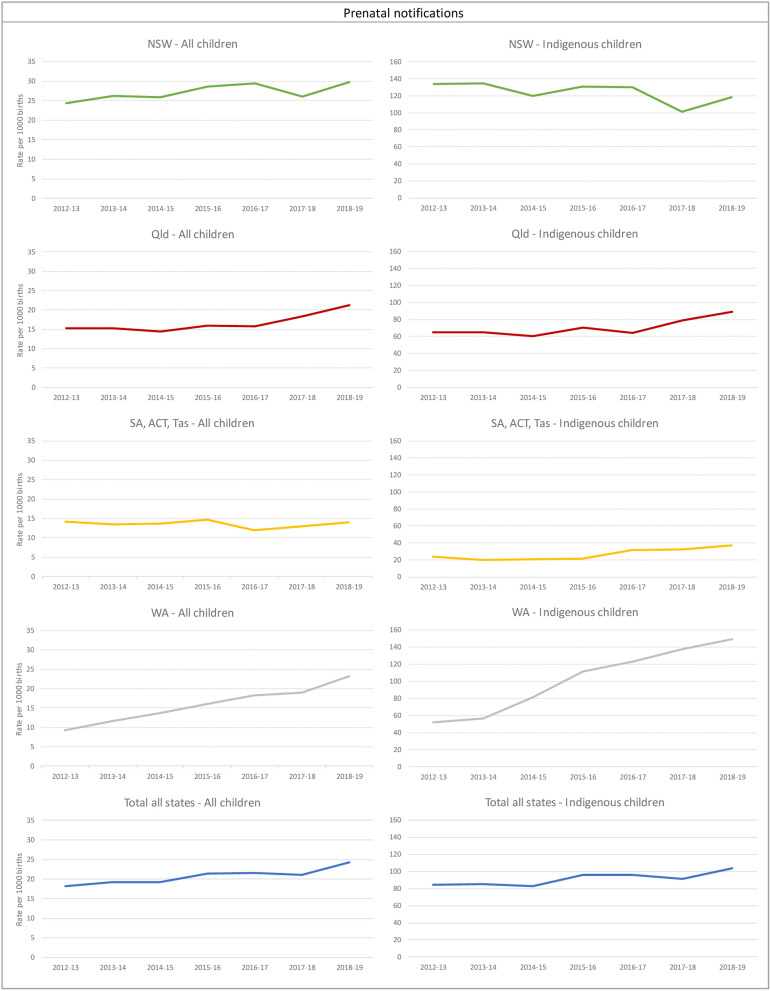
Children with prenatal notifications. Number and rate per 1000 births a) rates all children b) rates Aboriginal and Torres Strait Islander children. *Note:* NSW=New South Wales; Vic=Victoria; QLD=Queensland; WA=Western Australia; SA=South Australia; TAS=Tasmania; ACT=Australian Capital Territory; NT=Northern Territory. a: Y axes are presented in different scales for All children and Aboriginal and Torres Strait Islander children rates given the large difference. b: NT did not report prenatal notifications. c: Data are not comparable across jurisdictions due to differences in polices and data collection and report processes in each jurisdiction on notifications, investigations and substantiations. d: Given concerns on comparability on children with notifications between jurisdictions, each state is presented in a separate line chart.

From 2012-13 to 2018-19, NSW, WA and Qld (the three Australian jurisdictions who collect and provided approval for state-identified data on prenatal notifications) all three experienced increases in the rates of children with prenatal notifications to child protection services (Figure 1), with NSW accounting for over half of all children with prenatal notifications. The trend analysis showed a 3% increase in the rate per year for NSW (IRR:1.03 (1.02–1.03), a 6% increase per year for Qld (IRR:1.06 (1.04–1.07), and a 15% increase per year for WA (IRR:1.15 (1.13–1.17). Conversely, SA, ACT and Tas (which were combined) remained relatively unchanged (IRR:0.99 (0.97–1.01)).

The rate of children with prenatal notifications in 2018-19 overall in Australia was 24.4 per 1000 births, representing a total of 5384 children. Across Australia, there was an overall 4% increase per year (IRR: 1.04 (1.04–1.05)) in the rates of children with prenatal notifications; if we exclude NSW this rate was a 7% increase per year (IRR: 1.07 (1.06–1.07)).

Nationally, the rates of Aboriginal and Torres Strait Islander children with prenatal notifications in 2018-19 (103.7 per 1000) were over 4 times higher than the overall rate and had increased by 3% per year since 2012-13 (IRR: 1.03 (1.02–1.04)). There were 1194 Aboriginal and Torres Strait Islander children with prenatal reports in 2012-13, which increased to 1718 in 2018-19. Of the three jurisdictions who collect and provided approval for state-identified data on prenatal notifications, WA and Qld saw increases in rates of children with prenatal notifications for Aboriginal and Torres Strait Islander children at 19% and 5% respectively. SA, ACT and Tas combined had an 11% increase per year. NSW was the only state that showed a reduction in children with prenatal notification rates, decreasing by 3% per year (IRR: 0.97 (0.96–0.98)).

### Substantiations of Prenatal Notifications

On average, nationally, approximately 33% of children with prenatal notifications were substantiated. Across Australia, in 2018/19 1529 children had a substantiated prenatal notification. The primary type of substantiated maltreatment for children with prenatal notifications was neglect (59%) followed by emotional abuse (21%).

**Figure 2.**
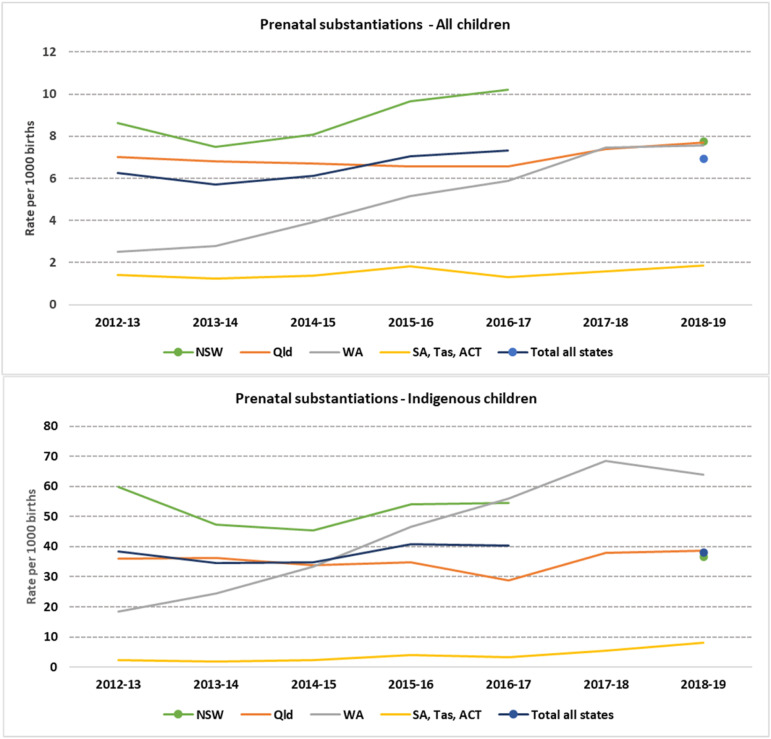
Children with prenatal substantiations. Rates per 1000 births a) all children b) Aboriginal and Torres Strait Islander children. *Note:* NSW=New South Wales; Vic=Victoria; QLD=Queensland; WA=Western Australia; SA=South Australia; TAS=Tasmania; ACT=Australian Capital Territory; NT=Northern Territory. a. NSW incorporated changes in their data collection system in 2017-18, with missing records for substantiations for that period. This had impacted in values for ‘Total all states’ for the period 2017-18, so that year was excluded. NSW data was available again for the period 2018-19, values for NSW and ‘Total all states’ are represented as a dots in the figure. b: Y axes are presented in different scales for Overall and Aboriginal and Torres Strait Islander children rates given the large difference. c: NT did not report prenatal substantiations. d: Data are not comparable across jurisdictions due to differences in polices and data collection and report processes on notifications, investigations and substantiations.

NSW accounted on average for more than half of the total number of children with substantiated prenatal notifications. In 2012-13, while NSW and Qld had the highest rates of children with substantiated prenatal notifications per 1000 births, at 8.6 (*N* = 846) and 7.0 (*N* = 443) per 1000 respectively, WA and the combined SA, Tas and ACT rates were much lower (2.5 -N = 85- and 1.4 per 1000 -N = 45-, respectively) (Figure 2). However, by the end of the study period (2018-19) this had changed: WA had increased its rate to be more in line with the NSW and Qld rate of children with prenatal substantiation of approximately 7 per 1000 births. Of the three jurisdictions who collect and provided approval for state-identified data on prenatal notifications WA was the only jurisdiction showing a significant increase in the rate of children with prenatal substantiations over time, at a rate of 21% per year between 2012-13 and 2018-19, when reached to 249 children. The combined SA, Tas and ACT rates remained low over the whole study period.

The rate of substantiations for Aboriginal and Torres Strait Islander children with prenatal notifications in NSW was 40% lower in 2018-19 (36.6 per 1,000, *N* = 246) compared to 2012-13 (59.8 per 1,000, *N* = 319) (Figure 2). However, the opposite trend was seen in WA, which started with a low rate in 2012-13 (18.3 per 1,000, *N* = 38) and reached a rate of 63.9 per 100 in 2018-19 (*N* = 151). This is equivalent to a yearly increase of 23% over that period (IRR: 1.23 (1.18–1.27)). Even though the combined rate of children with substantiations for SA, Tas and ACT was much lower compared to the other jurisdictions, combined they showed the highest increase on the rates for substantiated Aboriginal and Torres Strait Islander children with prenatal notifications within the period under study, at 29% per year (IRR: 1.29 (1.1–1.5)).

### Notifications of Infants (< 1 year old)

**Figure 3.**
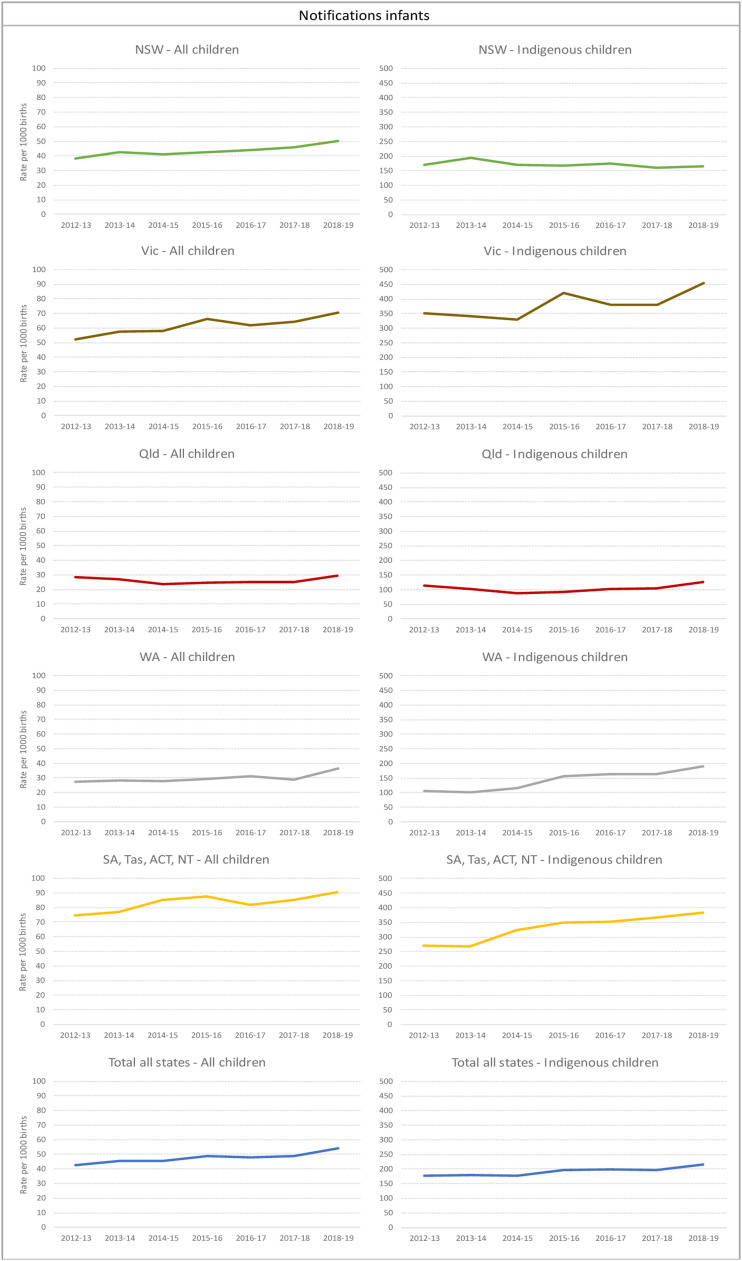
Children with notification as infants. Rates per 1000 children a) all children b) Aboriginal and Torres Strait Islander children. *Note:* NSW=New South Wales; Vic=Victoria; QLD=Queensland; WA=Western Australia; SA=South Australia; TAS=Tasmania; ACT=Australian Capital Territory; NT=Northern Territory. a: Y axes are presented in different scales for Overall and Aboriginal and Torres Strait Islander children rates given the large difference.b: Data are not comparable across jurisdictions due to differences in polices and data collection and report processes on notifications, investigations and substantiations.c: Given concerns on comparability on children with notifications between jurisdictions, each state is presented in a separate line chart.

Similar to prenatal notifications, we found increased rates of children with notifications as infants to child protection services across most jurisdictions (Figure 3). The overall Australian rate increased from 42.8 per 1000 children in 2012-13 (*N* = 13,018) to 54.3 per 1000 children in 2018-19 (*N* = 16,438), a 3% per year increase (IRR: 1.03 (1.03–1.04)). Rates in 2018-19 were highest in SA, Tas, ACT and NT (combined) (90.5 per 1,000, *N* = 3003), followed by Victoria (70.7 per 1,000, *N* = 5526), NSW (50.0 per 1,000, *N* = 4931) and WA (36.6 per 1,000, *N* = 1203). Qld was the only state that showed no significant increase in rates of children with infant notifications, reaching to 1775 infants in 2018-19.

Also similar to the proportion of children with prenatal notifications substantiated, 32% of children with infant notifications were substantiated on average. The primary type of substantiated maltreatment for infants in 2017-18 was emotional abuse (55%), followed by neglect (22%) and physical abuse (18%).

Nationally, the rate of Aboriginal and Torres Strait Islander children with infant notifications was almost four times higher than the overall rate. There was a rise in Aboriginal and Torres Strait Islander children with infant notification from 177 per 1000 in 2012-13 to 215.8 per 1000 in 2018-19 (Figure 3). The total number of Aboriginal and Torres Strait Islander children with at least one notification rose from 2999 children in 2012-13 to 4206 in 2018-19. In 2018-19, the highest rates were found in Victoria (453.3 per 1,000, *N* = 685) which had a 4% increase per year since 2012-13, and in SA, Tas, ACT and NT (combined) (382.8 per 1,000, *N* = 1253) with a 6% increase per year over the study period. In 2012-13, WA had the lowest rate of Aboriginal and Torres Strait Islander children with infant notification (105 per 1,000, *N* = 219), however, this increased to almost 200 per 1000 by 2018-19 (an average increase of 12% per year, *N* = 451).

### Infants (<1 year old) Admitted into OOHC

**Figure 4.**
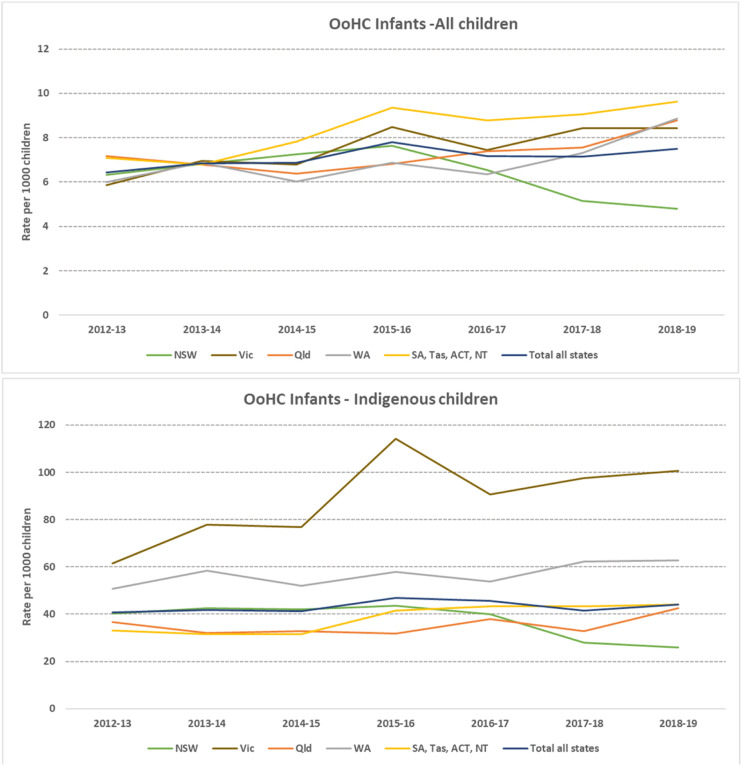
Infants admitted to out-of-home care placements. Rates per 1000 children a) all children b) Aboriginal and Torres Strait Islander children. *Note:* NSW=New South Wales; Vic=Victoria; QLD=Queensland; WA=Western Australia; SA=South Australia; TAS=Tasmania; ACT=Australian Capital Territory; NT=Northern Territory.a: Y axes are presented in different scales for Overall and Aboriginal and Torres Strait Islander children rates given the large difference. b: Data are not comparable across jurisdictions due to differences in polices and data collection and report processes on notifications, investigations and substantiations.

All jurisdictions had increases in the rates of infants admitted into OOHC, with the exception of NSW which saw a decrease to 474 infants in 2018-19 (IRR: 0.95 (0.94–0.97)) (Figure 4). Rates of infants entering care in Australia increased overall by 2% per year to 2268 infants in 2018-19 (IRR: 1.02 (1.01–1.03)), with Victoria having a 6% increase per year (IRR: 1.06 (1.04–1.07)), SA, Tas, ACT and NT (combined) also having a 6% increase per year (IRR: 1.06 (1.03–1.08)), and WA having a 5% increase per year (IRR: 1.05 (1.03–1.08)).

The rates for Aboriginal and Torres Strait Islander infants entering care were substantially higher at almost six times the overall rate, with 44 per 1000 Aboriginal and Torres Strait Islander infants entering care in 2018-19, a total of 859 infants. There was variability between jurisdictions in the trends in Aboriginal and Torres Strait Islander infants entering care over the study period (Figure 4). NSW experienced a 7% per year reduction in Aboriginal and Torres Strait Islander infants entering OOHC (IRR: 0.93 (0.91–0.95)) to 175 infants in 2018-19. The other states overall saw a 5% increase (total excluding NSW- IRR: 1.05 (1.03–1.06)). Victoria saw the largest increase (IRR: 1.07 (1.03–1.11)) in Aboriginal and Torres Strait Islander infant OOHC admissions, increasing to 100.6 per 1000 infants in 2018-19 (N= 152). SA, Tas, ACT, NT (combined) also increased by 7% per year (IRR: 1.07 (1.03–1.10)) to 44 per 1,000, which represented 144 infants.

### Time to Admission into OOHC

Nationally there has been a rise in the number of admissions to OOHC occurring in the first week following birth, with 634 newborn babies (0–7 days old) removed in 2018-19 and a further 385 removed within 8–31 days of their birth. This means that of the 2268 infants (<1 year) admitted to OOHC in 2018-19, one in four (28%) were removed in the first week after their birth and 45% within the first month. WA, Qld and SA, Tas, ACT, NT (combined) had a slightly higher proportion of children admitted into OOHC as infants removed in their first week of life compared to the national levels, at 35%, 33% and 31%, respectively (as stated, NT, Vic, ACT and SA don’t conduct investigations pre-birth). In 2012-13, nationally 26% of children who were admitted into care as infants were removed within one week after birth. WA (34%), Qld (33%) and NSW (29%) had a higher proportion of infants admitted into OOHC removed in the first week from birth than national levels, while Vic (13%) and SA, Tas, ACT, NT (17%, combined) showed lower proportions. Between 2012-13 and 2018-19, only NSW showed a decrease in the proportion of children entering care as infants removed in the first week of life. Conversely, Victoria and SA, Tas, ACT, NT (combined) more than doubled in the same period the percentage of infants who were admitted to care and were removed within the first week following birth.

## Discussion

This is the first study to use national unit record data to examine the patterns of child protection involvement in the lives of pregnant women/expectant parents and their infants in Australia. By examining such data, we were able to identify a number of similarities and differences among jurisdictions that collected the data being examined and provided approval for state-identified data.

Because NSW is the most populous jurisdiction and accounts for around half of all the rates reported, it has a disproportionate influence on the national rates. Examining individual jurisdictional rates and patterns is therefore particularly important. Following are the major patterns identified, noting there are limitations as not all jurisdictions collected the data and/or provided approval for state-identified data.

### Patterns of Prenatal and Postnatal Reports

Across Australia over the seven years to 2018-19, we have seen increases (4% per year) in the rate of children with prenatal notifications to child protection services. The three jurisdictions who collected and provided approval for state-identified data (WA, Qld and NSW) showed an increase in the rate of children with prenatal notifications in that period, at 15%, 6% and 3% per year respectively. Victoria did not report on the number children with prenatal notifications, and rates were stable in the SA, ACT and Tas combined. Over the same period, there was also an increase in the rate of children with infant (<1 year) notifications to child protection systems across Australia, with a 3% increase overall per year. Most jurisdictions had an increase in their rate of children with infant notification, with Qld the only state showing no significant change over time. The highest rate of children with infant notification over the study period were in the combined SA, Tas, ACT and NT, followed by Victoria.

### Patterns of Children with Substantiated Reports

Approximately 1 in 3 of both children with prenatal and infant notifications were substantiated. For both, the most common reasons were emotional abuse and neglect, but they differed in proportions: 59% of children with substantiated prenatal reports were for neglect, whereas 55% of children with substantiated infant reports were for emotional abuse. The infant reports are more in line with the overall proportions found in national child protection reporting, with emotional abuse at 54% and neglect at 21% (AIHW, 2021). NSW, Qld and WA had the highest rate of children with substantiated prenatal notifications in 2018-19, noting they were the only three jurisdictions who collect prenatal notification data and provided approval for state-identification. WA increased its rate of children with substantiations over recent years to be at a similar level to NSW and Qld.

### Patterns of Infant Entries to Care

All jurisdictions showed 5–6% increases in their rates of infant entries to care over the study period, except for NSW where care entries decreased. Increasingly infants were removed sooner after their birth, with almost all jurisdictions showing an increase in the proportion of infants removed one week after birth.

### Aboriginal and Torres Strait Islander representation in reports and removals

A significant over-representation of Aboriginal and Torres Strait Islander families was evident in all parts of the child protection system over the study period: Aboriginal and Torres Strait Islander families and infants were overwhelmingly over-represented in the rates of children with prenatal and postnatal notifications and substantiations, and in the admission to care of children under 1 year-old. However, there were notable variations by jurisdiction who provided state-identified data. While NSW had the highest rates of children with prenatal and infant notifications and substantiations in the earlier years of the study, they had been largely over-taken by the increasing rates in WA, who had started with the lowest rate in 2012-13.

NSW was the only state, of the three that collected prenatal notifications and provided approval for state-identified data, that showed a reduction in the rate of Aboriginal and Torres Strait Islander children with prenatal notification. Victoria had the highest rate of Aboriginal and Torres Strait Islander children with notifications as infants (453 per 1000 children) in 2018-19, and WA had the highest increase per year in the rate of Aboriginal and Torres Strait Islander children with infant notifications, at 12% per year. Nationally, Aboriginal and Torres Strait Islander infants were placed in out-of-home care at six times the overall rate. All states except NSW saw an increase in rates, despite removals into out-of-home care now recognised as a key *Closing the Gap* indicator and target area for reduction (Commonwealth of Australia, 2020).

### Jurisdictional Patterns

In England Bunting et al. (2017) utilised UK data to compare jurisdictional trends across England, Scotland, Wales and Northern Ireland finding variability in children protection involvement across jurisdictions. Similarly in Australia we are seeing state jurisdictional variability. NSW has experienced reductions in recent years in both the overall and Aboriginal and Torres Strait Islander rate of infant entries to care. They have also seen reductions in the rates of Aboriginal and Torres Strait Islander children with prenatal reports and substantiations and in the rates of children with infant notifications. Even with these changes, NSW has the highest rate of children with prenatal notifications and relatively low rates of infant entries to care compared to other states. Because NSW is the most populous jurisdiction and accounts for around half of all the rates of children reported, these reductions have a disproportionate influence on the national rates. A proportion of the NSW reductions in children with substantiations may be due to a new client management system that was introduced in 2017-18 (Australian Institute of Health and Welfare, 2021) which has impacted reporting of data. Indications from the most recent Child Protection Australia Report (2021) that substantiated notifications are increasing in NSW, particularly amongst Aboriginal and Torres Strait Islander families.

In WA, total population and Aboriginal and Torres Strait Islander children with prenatal reports and substantiations increased, as did overall infant care entries, while Aboriginal and Torres Strait Islander children with care entries remained stable. The increase in children with notifications could be driven by agencies (health and child protection) working together through a collaborative agreement to maximise early opportunity for planning and safeguarding of newborn infants.

While Victoria does not record prenatal reports, they had the highest and increasing rates of children with infant notifications and care entries, and a much higher rate of Aboriginal and Torres Strait Islander children with care entry than other jurisdictions. Qld alternatively had increases in children with prenatal notifications with high levels of substantiated notifications but had a lower rate of children with infant notifications, compared to other jurisdictions. Qld had increasing overall admissions into out-of-home care but, unlike Victoria, they did not have a significant rise in Aboriginal and Torres Strait Islander infant admissions into care.

The combined ACT, Tas and SA rates of children with prenatal notifications and substantiations were lower than the other jurisdictions, while their rates of children with infant notifications and care entry were higher (although not for Aboriginal and Torres Strait Islander infants). Given the requirement to present these jurisdictions in combination, the opportunities to attribute policy and practice impacts are limited. NT with small numbers and a lack of prenatal reports also limit the opportunity to investigate the trends. SA do not have the authority under legislation to formally report prenatal notifications for an investigative response, although SA are still able to provide a service delivery response.

While there are consistencies across states in regard to child protection prenatal and infants’ responses, there are also differences. In states such as NSW and WA, statutory reporting of child protection concerns can occur before birth (New South Wales Government, 2021; WA Health and Department for Child Protection and Family Support, 2020). The aim is to facilitate assistance and support to expectant parents in order to maximize the promotion of safety and wellbeing of the mother prenatally and subsequently the infant. However, in Victoria the Department of Health and Human Services do not receive prenatal statutory reports but can, with the mother’s consent, be referred for support. Therefore, in Victoria the number of prenatal notifications to the child protection agency is not collected or monitored and there is no investigation or determination of substantiated reports. Further research is required to look at the differential effects of the Victorian policy response as opposed to the responses by other states in which there are pre-birth statutory responses. Given the high level of infant removals in Victoria, key questions include whether the lack of statutory response prior to birth reduces the opportunities for intensive family support intervention pre-birth. Or whether the statutory responses pre-birth in other states means that women and families are under pressure to adhere to safety plans and attending services. Further evidence is required to understand the pathways and the effectiveness of different interventions and support services being offered and the impact on children and their families. Building this evidence is useful internationally for countries who are determining their perinatal responses to safety concerns during pregnancy and following birth.

## Implications for Policy and Practice

The rise in the rate of children with substantiated notifications in the majority of jurisdictions appears to be associated with the rise in rates of infants admitted into OOHC, with the exception of NSW which saw a decrease. Australian rates of infants entering care increased by 2% per year with most states showing a 4–6% increase per year. The high level of infant removals which occur in the first week (28%) and within the first month (45%) point to the importance of pre-birth planning and support for expectant families. The need for alternative pathways to removals such as supported placements for mothers and infants while safety is being worked towards needs to be part of a coordinated strategy for varying levels of risk. This indicates that there is an increasing amount of pre-birth work now required in maternity settings across Australia to assess health and safety risks for pregnant mothers and their unborn children, and demand for supportive interventions to address them.

A challenge in investigating Australian rates of children with prenatal and infant child protection involvement is that while there are nationally consistent definitions and specifications in the Child Protection National Minimum Data Set, there are jurisdictional policy and practice differences across states and territories (Australian Institute of Health and Welfare, 2020). These differences can impact on reported data and therefore comparability between states is difficult. Despite these limitations few countries consistently collect national child protection data. The use of child protection data is important in identifying emerging trends, ongoing child protection monitoring and interventions and determining implications for policy and practice within and across jurisdictions. These findings suggest increasing pressure on maternity services to assess families in regard to health and safety concerns and provide or refer families to support services. Given the high level of Aboriginal and Torres Strait Islander families and children over-represented in child protection notifications both pre- and post-birth, there is an urgent need to ensure Aboriginal and Torres Strait Islander community driven and culturally embedded services are available for Aboriginal and Torres Strait Islander pregnant women and families. This has also been called for internationally: Canada and New Zealand have also reported high rates of first nations child protection interventions during pregnancy and infancy (Fallon, 2021; Office of the Children’s Commissioner, 2020).

Given the rising rate of children with prenatal notifications there is demand for specialist expertise working with pregnant mothers and parents who have mental health, substance use or are experiencing family violence, which has been recognised by clinicians and researchers (Meiksans et al., 2021). It has also been reported that there has been a rise in complexity of cases which is challenging for those working in siloed service areas and the need for multidisciplinary and multiagency strategies is imperative to ensure complex needs of families can be met (Wise & Corrales, 2021). Most states do have pre-birth planning processes with bilateral agreements/schedules in place between Health and Child Protection that facilitates pre-birth planning meetings and, in the case of WA, the use of Independent Facilitators at these meetings (Department of Communities, 2021); (NSW Government, 2021); (Victorian Health and Human Services, 2021b). It is recognised that these processes have commenced at different times across jurisdictions and may have led to increased visibility in the pre-birth space as well as improved data collection and reporting systems. The challenges in Australia and internationally in the area of pre-birth planning with families is the short timeframe to implement support and interventions and this gives further imperative for multi-agency strategies to develop timely responses in collaboration with parents. Given the trend in most states of increasing infant removals there is the need to provide options post-birth for supported placements for parent/s with the infant if there are ongoing risks that need to be managed. In scenarios where the risks are too high for the baby to remain with the parent/s, family conference meetings are essential to discuss placement with extended family or other alternatives, with contact visits arranged prior to removal and ongoing family reunification services available. Many states have commenced or extended pre-birth planning in their programs of family group conferencing or Aboriginal family led decision-making which encourages family participation in safety planning and supported placements with kin.

With rising numbers of pregnant women and families reported prenatally and during infancy, greater evidence around the effectiveness of policies, processes interventions and the long-term outcomes for children and families is required both in Australia and internationally to provide evidence-based responses to safety concerns. Canada has debated the efficacy of birth alerts with variation in approaches among provinces (Sistovaris et al., 2021). In terms of family support there are promising programs that are indicating effectiveness for families with complex needs such as Parenting under Pressure, Pause – creating space for change, Multisystemic Therapy, and Trauma Adapted Family Connections (Breiner et al., 2016; Collins et al., 2011). However, further research is required to determine for which groups strategies and interventions are most effective and there is still the need for culturally informed strategies and interventions for Aboriginal and Torres Strait Islander and First Nations families and communities (Chamberlain et al., 2022). Chamberlain et al. (2022) call for a focus on: prevention with a redesign of maternity services so parents have access to culturally responsive, trauma integrated support; partnership with Aboriginal communities for culturally embedded care; participation with parents, families and communities at the centre of child protection decision-making; and connection following removals to ensure ongoing relationships with parents and reunification efforts. Research is also required into the multidisciplinary decision-making, assessment and interventions that families are being subject to and that families have a voice in this process. Evidence based policy, processes, support and interventions are essential to improve safety and wellbeing outcomes for infants and their families and ensuring these are culturally embedded is imperative given the high rates of infants and families impacted across Aboriginal and Torres Strait Islander communities.

## Note

1. This includes pregnant persons who do not identify as a woman.

## Appendix

**Table A1.** Trend analysis by state and territory for children with prenatal child protection notifications and substantiations, and infants notifications and OOHC placements.

	Prenatal		Infants	
	Notifications	Substantiations	Notifications	OOHC
	IRR (95%CI)	IRR (95%CI)	IRR (95%CI)	IRR (95%CI)
Overall				
NSW	1.03 (1.02–1.03)*	1.01 (0.99–1.02)	1.04 (1.03–1.04)*	0.95 (0.94–0.97)*
Vic^a^			1.04 (1.04–1.05)*	1.06 (1.04–1.07)*
QLD	1.06 (1.04–1.07)*	1.02 (1.00–1.03)	1.00 (0.99–1.01)	1.04 (1.02–1.06)*
WA	1.15 (1.13–1.17)*	1.21 (1.18–1.25)*	1.04 (1.03–1.05)*	1.05 (1.03–1.08)*
SA, TAS, ACT & NT^a^	0.99 (0.97–1.01)	1.05 (0.99–1.10)	1.03 (1.02–1.03)*	1.06 (1.03–1.08)*
Total NSW, Vic, QLD & WA^a^	1.05 (1.04–1.05)*	0.99 (0.98–1.00)*	1.04 (1.03–1.04)*	1.02 (1.01–1.02)*
TOTAL (all states)	1.04 (1.04–1.05)*	0.99 (0.98–1.00)	1.03 (1.03–1.04)*	1.02 (1.01–1.03)*
Total excluding NSW	1.07 (1.06–1.07)*	1.07 (1.05–1.08)*	1.03 (1.03–1.04)*	1.05 (1.04–1.06)*
Aboriginal and Torres Strait Islander children				
NSW	0.97 (0.96–0.98)*	0.95 (0.93–0.97)*	0.98 (0.97–1.00)*	0.93 (0.91–0.95)*
Vic^a^			1.04 (1.03–1.06)*	1.07 (1.03–1.11)*
QLD	1.05 (1.03–1.08)*	1.01 (0.98–1.03)	1.02 (1.00–1.04)*	1.03 (1.00–1.05)
WA	1.19 (1.16–1.22)*	1.23 (1.18–1.27)*	1.12 (1.09–1.14)*	1.03 (1.00–1.06)
SA, TAS, ACT & NT^a^	1.11 (1.05–1.17)*	1.29 (1.10–1.50)*	1.06 (1.05–1.08)*	1.07 (1.03–1.10)*
Total NSW, Vic, QLD & WA^a^	1.03 (1.02–1.04)*	0.98 (0.96–0.99)*	1.02 (1.02–1.03)*	1.00 (0.99–1.02)
TOTAL (all states)	1.03 (1.02–1.04)*	0.98 (0.97–1.00)*	1.03 (1.03–1.04)*	1.01 (1.00–1.02)
Total excluding NSW	1.10 (1.09–1.12)*	1.08 (1.06–1.10)*	1.06 (1.05–1.06)*	1.05 (1.03–1.06)*

**p* < 0.05.

NSW = New South Wales; Vic = Victoria; QLD = Queensland; WA = Western Australia; SA = South Australia; TAS = Tasmania; ACT = Australian Capital Territory; NT = Northern Territory.

a: Victoria and NT are not included in the prenatal analysis.
